# Association between Compliance with COVID-19 Restrictions and the Risk of SARS-CoV-2 Infection in Poland

**DOI:** 10.3390/healthcare11060914

**Published:** 2023-03-22

**Authors:** Karolina Hoffmann, Michał Michalak, Aleksandra Bońka, Wiesław Bryl, Wojciech Myśliński, Magdalena Kostrzewska, Dorota Kopciuch, Tomasz Zaprutko, Piotr Ratajczak, Elżbieta Nowakowska, Krzysztof Kus, Anna Paczkowska

**Affiliations:** 1 Department of Internal Diseases, Metabolic Disorders and Arterial Hypertension, Poznan University of Medical Sciences, 60-572 Poznań, Poland; 2 Department of Computer Science and Statistics, Poznan University of Medical Sciences, 61-701 Poznań, Poland; 3 Department of Pharmacoeconomics and Social Pharmacy, Poznan University of Medical Sciences, 61-701 Poznań, Poland; 4 Department of Internal Disease, Medical University of Lublin, 20-059 Lublin, Poland; 5 Department of Pulmonology, Allergology and Pulmonological Oncology, Poznan University of Medical Sciences, 61-701 Poznań, Poland; 6 Department of Pharmacology and Toxicology Institute of Health Sciences, Collegium Medicum, University of Zielona Góra, 65-417 Zielona Góra, Poland

**Keywords:** compliance, COVID-19, personal protective equipment, physical distancing, restrictions

## Abstract

During the coronavirus disease 19 (COVID-19) pandemic it has become very important to comply with preventive measures. We aimed to assess compliance with applicable restrictions and to explore the links between the level of compliance and the risk of COVID-19. This cross-sectional study included Polish adults who were asked to complete a validated questionnaire. The study period was from 1 November 2020 to 31 January 2021 and a computer-assisted web interview method was chosen to perform the survey. The study involved 562 women and 539 men. COVID-19 was reported in 11.26% of participants. A good level of compliance with the sanitary restrictions was reported for 38.87% of participants, an average level of compliance for 47.96%, and a low level of compliance for 13.17%. A reduced risk of COVID-19 was associated with the following preventive measures: regular use of protective masks, social and physical distancing in public places, regular use of hand sanitizers with high ethanol content, and the use of disposable gloves in public places. Our survey revealed satisfactory public compliance with the pandemic restrictions. Sanitary and epidemiologic measures to prevent the pandemic were shown to be adequate and effective.

## 1. Introduction

Since its outbreak in 2020, the coronavirus disease 19 (COVID-19) pandemic has radically changed the lives of people across the globe. The disease is caused by a novel coronavirus originally named 2019 novel coronavirus (2019-nCov). However, on 11 February 2020, the name was officially changed to severe acute respiratory syndrome coronavirus 2 (SARS-Cov-2) [1]. The first case of SARS-Cov-2 infection was detected at the end of 2019 in Wuhan (China) [1,2]. Patients with COVID-19 are the primary source of infection, and the virus exhibits the highest infectivity 1 to 3 days from onset of the first symptoms [1,2].

### 1.1. The Epidemiological Situation in Poland and in the World during the Second Wave of the COVID-19 Pandemic

On 10 March 2023, there were 681,301,970 confirmed cases of COVID-19 worldwide, and death occurred in 6,810,359 patients. In Poland, there were 6,448,577 total cases and 119,016 total deaths [3]. At the time of the second wave of the pandemic in Poland (in late 2020 and early 2021), there were 90.2 million cases of infection and 1.9 million deaths globally [3]. In Poland, the respective numbers were 991,000 infections and over 17,000 deaths (as of 30 November 2020). Importantly, 11,519 patients with COVID-19 died in November 2020, marking the highest peak in mortality since the onset of the pandemic in Poland. The factors contributing to such a high death toll included the lack of adequate treatment for comorbidities [4,5]. The mortality rates reached over 7% in patients aged 60 to 80 years and nearly 20% in those older than 80 years. The disease soon appeared to have an unpredictable course and a complex pathogenesis, and there was no effective treatment. Moreover, some people denied the very existence of the virus. It quickly became clear that there was a strong need to develop a strategy to combat COVID-19 as soon as possible [6]. The isolation of infected patients and the reduction in virus transmission emerged as some of the most critical steps in the fight against the pandemic [7]. The alpha variant of SARS-CoV-2 (the so-called British variant, B.1.1.7) was predominant during the second wave of the pandemic in Poland. Its infectivity was estimated to be 50% to 70% higher than that of the original SARS-CoV-2 strain. In early 2021, it was also the most widespread variant of the virus in the world [8].

### 1.2. The Need for an Effective Strategy to Fight the COVID-19 Pandemic—The Polish Experience

To control the spread of the virus, it was necessary to implement preventive sanitary measures. On 20 March 2020, the Minister of Health introduced a state of epidemic throughout the country [9]. Specific restrictions based on the new legal regulations came into effect [10]. Asymptomatic patients who tested positive for SARS-CoV-2 were subject to a 10-day isolation. For symptomatic individuals with COVID-19, a general practitioner extended the isolation period as follows: 10 days from the onset of symptomatic illness and at least 3 asymptomatic days (no fever or respiratory symptoms). Moreover, a mandatory quarantine was imposed on people crossing the border of the Republic of Poland or an external border of the European Union using organized transport, as well as on those referred for COVID-19 testing by a general practitioner. People who had been in close contact with a person diagnosed with COVID-19 were also subject to quarantine. Moreover, people who lived with a person infected with SARS-CoV-2 (as confirmed by a positive diagnostic test) were required to undergo quarantine from the date the housemate received the test results up to 7 days after he or she ended the quarantine. Only medical staff and people vaccinated against COVID-19 were exempt from quarantine. Individuals in isolation or under quarantine were not allowed to leave their homes, except in certain circumstances, such as a medical visit [10].

In Poland, the second wave of the COVID-19 pandemic began in the middle of October 2020 and ended in late January 2021. Based on the Council of Ministers Regulation of 23 October 2020, an obligation to wear masks in public areas was introduced as of 24 October 2020. Moreover, limitations were imposed on the number of people permitted to be in retail stores, depending on the size of the store. Restaurants, bars, gyms, fitness centers, swimming pools, and sanatoriums were closed. Finally, online learning was introduced for primary schools (grades 4–8) [11].

Owing to the rapid increase in the number of new COVID-19 cases, the existing restrictions were extended, and some new restrictions were introduced in Poland by the Regulation of 6 November 2020. The activity of public and nonpublic educational institutions was restricted, including primary schools (all grades), secondary schools, and higher education establishments. Theaters, cinemas, museums, art galleries, community centers, and music centers were closed; hotels became available only to guests on business trips; the operation of shopping malls and stores was limited; the number of people in stores with less than 100 m^2^ of floor space was limited to one person per 10 m^2^, and in those with more than 100 m^2^ of floor space, to 1 person per 15 m^2^. In churches, the limit was one person per 15 m^2^ of floor space [12].

On 28 November 2020, some restrictions were partially lifted because of the upcoming Christmas holidays [13]. A physical distance of 1.5 m from other people in public places was allowed. Bars and restaurants were permitted to offer takeaway and delivery services, while hairdressing and beauty salons were allowed to serve customers under a sanitary regime. Gatherings and meetings were limited to a maximum of five people, and sports events could be organized without an audience.

On 17 December 2020, restrictions applicable for the period from 28 December 2020 to 17 January 2021 were announced [14]. Ski lifts were closed, sports infrastructure was open for professional sports only, and a 10-day quarantine was mandatory for people coming to Poland by organized transport. On 11 January 2021, the existing restrictions were extended until 31 January 2021. The big news for the public was that the COVID-19 vaccination program would start on 15 January 2021 [15]. Medical staff (the so called ‘zero’ group) were vaccinated from 27 December 2020. Finally, the Polish National Vaccination Program was launched on 25 January 2021. The first age group offered free vaccination were seniors older than 70 years [15].

### 1.3. The Problem of Compliance with Pandemic Restrictions

Societies across the world had to face the new epidemiological situation. People responded to the restrictions in various ways. Generally, the degree to which a person follows the recommendations is known as “compliance” [16]. Factors influencing support or opposition to various restrictions imposed by governments during the COVID-19 pandemic have been discussed in the literature [17,18,19]. However, the data collected during this time should be carefully investigated to answer emerging questions about the most effective strategies to reduce the spread of viral respiratory diseases. A recently published analysis of data collected before the SARS-CoV-2 pandemic indicates that hand hygiene programs effectively reduce the spread of viruses, but the effectiveness of some epidemic restrictions, including wearing masks or N95/P2 respirators, is still debatable [20]. Jefferson et al. underlined that large pragmatic trials should be conducted to clearly demonstrate the most effective sanitary restrictions in various settings [20].

### 1.4. Study Aims

The scientific literature does not contain sufficient data on the impact of compliance with sanitary measures on the risk of COVID-19 in Poland. Therefore, in this study we aimed to assess compliance with the applicable restrictions during the second wave of the SARS-CoV-2 epidemic in Poland and to investigate the associations between compliance with sanitary measures and the risk of COVID-19.

## 2. Materials and Methods

### 2.1. Sample Composition

A formula for a finite population was used to calculate the sample size [21]. It was estimated that the required number of respondents was 1068. The following data were taken into account: an estimated number of 20 million adult Poles with access to the Internet [22], a fraction size of 50% (a standard if the fraction is unknown), and a permissible error (e) of 3%. All recruited subjects met the following inclusion criteria: they were of legal age, lived in Poland and could give their informed consent to participate in the study. Individuals who did not meet the inclusion criteria were excluded. A total of 1157 respondents (all who agreed to participate in the study) were taken into consideration. However, based on the inclusion criteria of the study and incompletely filled in questionnaires by 56 Poles, 1101 respondents were finally included in the study.

On 4 November 2020, the Bioethics Committee at the Poznan University of Medical Sciences (Poznań, Poland) confirmed that the study had no features of a medical experiment. Before enrolment, volunteers were instructed about the study goals and informed that they were allowed to withdraw their consent to participate in the survey at any time without providing a reason. The identity of the respondents was anonymous. The study did not violate the Personal Data Protection Act, as no personal data of the participants were collected.

### 2.2. Research Methods and Time Horizon

A prospective, cross-sectional web-based survey design was adopted. For the purpose of this study, a validated anonymous questionnaire was created using Google Forms, based on the scientific literature. The study was conducted from 1 November 2020 to 31 January 2021. During the state of epidemic in Poland, a computer-assisted web interview method seemed to be optimal for reducing the risk of infection among respondents. Social media (e.g., Facebook, Instagram, Linkedin) were used to distribute questionnaires. Questionnaires were also distributed electronically (by e-mail) throughout the country [23,24]. Two thousand e-mails were sent to Polish adults with a request to join the study voluntarily. The e-mail contained a link to the research questionnaire and all necessary information about the study’s purpose and rules of participation. The response rate was 55.05%. The Google forms platform recognized the IP address of the interviewee computer and, thus, prevented the same person from submitting multiple questionnaires. The study questionnaire contained mostly mandatory fields, which made it impossible to omit some answers. It was developed on the basis of a literature review, focused group discussions, and expert opinion. A pretest procedure on a representative sample of 300 respondents (150 women and 150 men) was used to validate the questionnaire. The internal consistency of the study tool was calculated with the use of the Cronbach’s α coefficient to determine correlations between individual items. This coefficient was 0.73 for the total score, indicating excellent internal consistency. Five national experts on infectious diseases checked and approved the final version of the research tool. The questionnaire comprised 41 questions grouped into four sections: characteristics of the respondents, self-reported health status during the pandemic, compliance with restrictions, and the course of SARS-CoV-2 infection among respondents. Supplementary Material file S1 includes the research tool. Based on the questions concerning compliance with COVID-19 preventive measures, a list was created in which each individual respondent scored points according to predefined criteria. The level of compliance with COVID-19 restrictions during the study period was assessed on the basis of responses to questions relating to: the use of a mask and/or a face shield to cover the mouth and nose in public places; hand washing with soap and water; use of hand sanitizers with high ethanol content; social distancing (minimum 1.5-m distance) in public places; and self-reported use of disposable gloves in public places. For the yes-no questions, four points were scored for a “yes” answer and 0 points for a “no” answer. For the multiple-choice questions, a Likert scale was implemented. The points on the scale indicated the level of agreement with a given statement, for example: 0 = “never”, 1 = “rarely”; 2 = “sometimes”, 3 = “often”, and 4 = “very often”.

The final score reflected the level of compliance with the restrictions. A score of 0 to 14 indicated a low level of compliance; 15 to 18, an average level of compliance; and 19 to 24, a good level of compliance. The datasets used and/or analyzed during the current study are available from the corresponding author on reasonable request.

### 2.3. Statistical Analysis

Counts and percentages were used to present categorical variables. The compliance value was presented as the median and interquartile range (Me, Q_1_-Q_3_), as well as numbers and percentages in cases where compliance was represented in terms of categories (e.g., Good, Average, Low). A linear regression analysis was performed to assess the relationship between compliance and selected parameters. For categorical variables, the coefficients of a specified level were compared to a reference level. In order to assess the relationship between the risk of COVID-19 and the analyzed parameters, a logistic regression model was used. The linear regression results were presented as coefficient values (coeff.) and their standard errors (SE), and the logistic regression results were presented as odds ratios (OR) and their 95% confidence intervals (95%CI). The models were determined twice: once as a univariate model and secondly as multiple models. The multiple models were assessed using a stepwise backward selection procedure. A test probability of a *p* value less than 0.05 was assumed to be significant. The Statistica data analysis software v. 13.3 (TIBCO Software Inc. 2017, Palo Alto, CA, USA; http://statistica.io accessed on 1 April 2021) was used to perform the statistical analysis.

## 3. Results

The survey included 1101 people (562 women and 539 men). Table 1 presents the sociodemographic characteristics of the study population. The attitudes of respondents to the pandemic restrictions are shown in Table 2, and the level of compliance with the restrictions is presented in Table 3. The multiple regression analysis revealed that compliance with COVID-19 restrictions was associated with age (Table 4). Respondents aged over 60 years showed the highest level of compliance. There was also a significant relationship between self-reported health status and compliance (Table 4). The level of compliance was lower among respondents who reported worse health status.

Most respondents were not subject to mandatory quarantine (74.93%). Of the remaining respondents, 21.89% underwent quarantine once, 2.82% underwent quarantine twice, and 0.36% underwent quarantine more than twice. No direct contact with a person diagnosed with COVID-19 was reported by 71.39% of respondents, and 54.77% of respondents reported no contact with a person suspected of SARS-CoV-2 infection or with infectious material.

SARS-CoV-2 testing was reported by 29.88% of respondents, of whom 62.31% (*n* = 205) tested negative and 37.69% (*n* = 124) tested positive for the infection. Of the 124 participants with a positive result (11.26% of the whole study group), hospitalization for COVID-19 was reported by only 2 (1.61%). Of all the individuals with COVID-19, 25.81% (*n* = 32) developed complications, including loss of smell (12.50%), headache (12.50%), fatigue (12.50%), chest pain (9.38%), back pain (9.38%), and poor concentration (9.38%).

The logistic regression model confirmed that compliance with pandemic restrictions reduced the risk of SARS-CoV-2 infection. Factors that were significantly associated with a lower risk of infection included the regular use of protective masks, social distancing in public places, regular use of hand sanitizers with high ethanol content, and the use of disposable gloves in public places (Table 5**)**.

## 4. Discussion

This innovative study conducted in Poland during the second wave of the COVID-19 pandemic provides reliable data on the attitude of Poles to the restrictions imposed by the government to limit the transmission of SARS-CoV-2. Currently, compliance with sanitary and epidemiological measures is being widely discussed in the context of a global strategy to combat SARS-CoV-2 [25]. However, there is a lack of sufficient data in the literature on the extent to which compliance with specific restrictions contributes to reducing the risk of SARS-CoV-2 infection. Our study showed that 86.83% of respondents had a good or an average level of compliance with the restrictions. Most of them reported covering the mouth and nose with a mask or a face shield in public places, as well as covering the mouth when sneezing or coughing. Moreover, our study showed that the regular use of protective masks significantly reduced the risk of SARS-CoV-2 infection. Wearing a mask is a critical sanitary measure applied worldwide to reduce the spread of COVID-19. However, it has not been in force since the very beginning of the pandemic [26,27,28]. The World Health Organization (WHO) initially recommended hand washing and disinfection as well as social distancing and isolation, while the use of facemasks was not indicated due to inconclusive evidence for its positive effect on reducing SARS-CoV-2 transmission [29,30]. However, in 2020, the droplet route of virus transmission was confirmed, supporting the health benefits of covering the mouth [31]. Following this, the recommendation to use a facemask was introduced in the same year [32]. As the presented study confirmed the positive role of the use of protective masks, it should be taken into account by policymakers whether this recommendation should be extended, if only in healthcare facilities. Furthermore, the obtained data may be useful in improving the strategy to prevent the spread of COVID-19 and other respiratory infectious diseases in the future. Previous studies have also suggested the reasonableness of introducing such a recommendation [33,34]. There has been a decline in the number of cases, including of influenza and parainfluenza, over the last two years [35,36].

In an observational study at two locations in Hawaii (downtown Honolulu business area and a tourist area of Waikiki), Tamamoto et al. [37] reported that 77% of subjects used facemasks correctly. The rate of public compliance with facemask use was significantly higher in Honolulu than in Waikiki (88% vs 66%; *p* = 0.0003; odds ratio [OR], 3.78; 95% CI, 1.82–7.85) [37]. In 2020, the International Citizen Project Covid-19 consortium conducted online surveys including 206 729 adults residing in nine low-income and middle-income countries. The study revealed that facemasks were used correctly by 32.7% to 99.7% of individuals [38]. Moore et al. [39] compared German and Australian respondents and revealed higher scores for wearing masks outside the home in the German sample.

Almost 95% of surveyed Poles reported a high frequency of hand hygiene, including hand disinfection in public places. Our analysis revealed that the regular use of hand sanitizers with high ethanol content significantly reduced the risk of SARS-CoV-2 infection. Hand washing and disinfection is one of the major preventive measures against COVID-19 [40,41]. Guzek et al. [42] studied protective behaviors in a sample of 2323 Polish students aged between 15 and 20 years. Female students were better educated in personal protection and hand hygiene (*p* < 0.05) and had a higher daily frequency of hand washing (*p* < 0.0001) than male students. Moreover, 68.2% of female students washed their hands whenever necessary, as compared with 54.1% of male students (*p* < 0.0001) [42].

Moore et al. [39] reported higher hand washing scores in the German sample than in the Australian sample. On the other hand, Australian participants reported significantly higher scores for the use of hand sanitizers and adherence to government recommendations. While Australians scored higher on using sanitizers, the German sample scored higher on hand washing. This suggests that both groups are equally aware of the need to cleanse the hands during the pandemic. In both samples, the hand washing scores were high [39]. Hand washing and the use of sanitizers have been reported to reduce the spread of SARS-CoV-2 since 2020 [43,44,45]. Experiences from the fight against COVID-19 can inspire future public health campaigns and show the public that high levels of hand hygiene can help solve the problem of the spread of infectious diseases. More research needs to be undertaken to choose the most effective, environmentally friendly and skin safe disinfectant.

In our study, 83.83% of respondents maintained social distance in public places. Social distance was shown to significantly reduce the risk of SARS-CoV-2 infection. The importance of this preventive measure for controlling the spread of the virus was already emphasized by the Centers for Disease Control and Prevention and WHO in 2020 [46,47]. The impact of physical distancing on reducing SARS-CoV-2 transmission was confirmed by Chung and Chan in a study using data from 17 countries [48]. Similar conclusions were reported by Bielecki et al. [49] in a comparative cohort study including young soldiers. The authors revealed that compliance with a social distancing recommendation could slow the spread of the coronavirus and prevent symptomatic COVID-19 [49]. In a Chinese study, 95.6% of the 2130 adults admitted to adhere to social distancing. Female respondents were more likely to do so than male respondents (OR, 3.12; 95% CI, 1.93–5.02) [50]. Geriatrics’ experts pointed out that little is known about the positive and negative consequences of social distancing in nursing homes [51]. Future research should focus on the well-being of older residents during lockdown due to the epidemic. This part of society, which already experiences loneliness at this stage of life, is particularly sensitive to further negative effects of social distancing imposed by the pandemic. Pandi-Perumal et al. even suggested changing the misleading term “social distancing” into “distant socializing”, pointing to the role of maintaining good, satisfying relationships even in times of epidemic [52].

In our study, respondents showed the lowest compliance with the recommendation to use disposable gloves during shopping. Nearly 70% of the respondents never used gloves or used them rarely. The use of disposable gloves in public places was also shown to significantly reduce the risk of SARS-CoV-2 infection [53,54]. However, this preventive measure has not been as well regulated by the authorities as other measures. The issue of wearing gloves in public has so far not been investigated in randomized controlled trials (RCTs). A similar methodological problem concerns gowns, face shields, and screening at entry ports [20]. Some institutions recommended the use of disposable gloves in public places [55]. However, it was also reported that such a strategy may be unsuitable for self-protection because it might be responsible for a higher risk of cross-transmission if precautions were not followed [56]. Moore et al. [39] showed that German and Australian respondents rarely wore gloves outside the house, and the rates were similar in both groups [39]. Another concern of wearing gloves in everyday life is the risk of allergic dermatoses. To prevent this, it is very important to share knowledge with the public about the different types of gloves and how to wear them correctly [57].

Our respondents differed in their attitudes to health protection measures: some judged them as adequate, while others considered them to be either too lenient or too strict. A report by the Public Opinion Research Centre (in Polish, Centrum Badania Opinii Społecznej) showed that 59% of Poles considered the coronavirus pandemic as an exceptional situation, while 32% noted that there have always been seasonal diseases and epidemics [58]. The remaining 9% of respondents had no opinion. Respondents who viewed the pandemic as exceptional were most often older, lived in large cities, and had a higher education level. Moreover, 78% of respondents reported that their neighbors complied with the restrictions, 18% estimated that most of their neighbors did not adhere to any restrictions, and 4% had no opinion on this issue [58]. These results are in line with our findings.

The multiple regression analysis showed that the level of compliance with the restrictions increased with the age of respondents. Higher compliance among older people may be explained by the fact that they may be more aware of chronic diseases contributing to a worse course of COVID-19. It is possible that respondents aged over 60 years are more afraid of a sudden deterioration in health than younger people are. Thus, compliance with recommendations is an important self-protection measure against COVID-19 among elderly people [59]. In a national survey conducted in Ireland, older age was also independently associated with compliance with local restrictions during the pandemic of COVID-19 [60]. Both the above-cited and our findings indicate the need to focus media campaigns on the role of the sanitary regime at less aware citizens, including younger people.

Interestingly, our statistical analysis showed a relationship between a worse self-reported health status and lower adherence to sanitary measures. A similar link was reported for patients with diabetes. This may be explained by the increasing lack of motivation in the face of a deteriorating health condition, as well as by the negative effect of the SARS-CoV-2 pandemic on numerous aspects of mental health [59,61].

In our study, SARS-CoV-2 infection was reported in only 11.26% of cases, and most respondents did not undergo mandatory quarantine. The low rate of infections may be explained by the fact that a large percentage of respondents showed a good or an average level of compliance with sanitary restrictions. Of note, after the second wave of the pandemic in our country, fewer people were sent on quarantine than before. Quarantine for the co-household members of a person infected with COVID-19 was no longer required [62]. From an economic point of view, quarantine can jeopardize employment relationships. Therefore, it was postulated that it should be limited only to direct contacts of infected individuals [63]. Using the synthetic control method, Zhu and Than [64] constructed a model of a hypothetical reality that contradicts the observed facts (a so-called counterfactual model) to compare home vs. centralized quarantine. They revealed that home quarantine is cheaper than centralized quarantine, while its effectiveness is similar (infection rate, 0.136% and 0.174%, respectively). The authors concluded that home quarantine will probably remain the preferred measure globally, especially in countries with limited financial resources [64].

Our survey revealed that the COVID-19 pandemic raised general awareness among Poles that compliance with sanitary and epidemiological measures is important. However, there is still a clear need to disseminate evidence-based information on the relationship between high levels of compliance and reduced risk of SARS-CoV-2 infection [65]. It is also important to improve individual and collective understanding of behaviors and attitudes towards the restrictions [66,67]. When people are convinced about the need to apply self-protection measures and avoidance strategies, their compliance level is high, as shown by Moore et al. [39]. It seems that educational campaigns and other ways to improve public compliance should be a key element in designing effective health communication about the COVID-19 pandemic and other health crises in the future [68]. As the end of the pandemic cannot be predicted, global efforts to overcome COVD-19 are still necessary.

### Study Limitations and Strengths

This study has several limitations. A computer-assisted web interview method was chosen to perform the survey, which contained only self-reported answers. However, the digital environment is a novelty that appears to be the primary method for data collection during an epidemic. Both social isolation and the growing fear of the real world during the pandemic have affected people’s lives. Our habits and lifestyles have evolved to become adapted to the digital environment. However, increased online activity is observed especially among young individuals. In our study group, 62.93% were younger than 40 years. In addition, online research does not allow random recruitment of respondents because of limited access to the Internet or the participant’s interest in the surveyed topic. However, it should be underlined that our online questionnaire contained mostly mandatory questions, which made it impossible to omit some answers, as is often the case with paper questionnaires. Moreover, the study used a proprietary, unverified questionnaire due to the lack of an appropriate research tool in the literature.

The present study is also limited by the fact that the level of compliance with the restrictions was assessed at only a single time point. We are aware that compliance may have changed over time and, thus, may have affected the risk of SARS-CoV-2 infection. Finally, the survey was conducted in a single country. Therefore, future studies are needed to provide insights into cross-country comparisons and the impact of social, political, and cultural differences on compliance with restrictions.

Another limitation is that some participants may have been healthcare professionals or had a healthcare professional in the same household, which may have affected compliance.

The strength of this study is its novelty. Further research is needed to investigate the impact of compliance with government regulations on the course of the SARS-CoV-2 epidemic. Further research is needed to investigate how compliance with government regulations could influence the SARS-CoV-2 epidemic. Follow-up studies should also determine whether Polish residents continue to apply protective strategies and adhere to restrictions. Our findings can provide the basis for healthcare policymakers and medical professionals to develop clear recommendations for the public and, thus, help improve compliance.

## 5. Conclusions

Our survey showed a satisfactory level of compliance with pandemic restrictions among Poles. There was a significant relationship between compliance and two factors: age and self-reported health status. The results confirmed that sanitary and epidemiological measures introduced by the Polish authorities were adequate. Wearing protective masks, social distancing strategies, hand disinfection, and the use of disposable gloves in public places significantly reduced the risk of SARS-CoV-2 infection. This is in line with the pandemic recommendations introduced by the WHO in 2020 and still applies today [69].

## Figures and Tables

**Table 1:** Sociodemographic characteristics of the study population (*n* = 1101).

Variable		Number of Respondents, n	Percentage of Respondents, %
Sex	Female	562	51.00
	Male	539	49.00
Age, y	18–24	411	37.33
	25–40	382	25.61
	41–60	206	18.71
	>60	102	9.26
Education	Primary	66	6.00
	Basic vocational	80	7.26
	High school	955	86.74
Place of residence	Village	266	24.16
	Town < 50,000 residents	169	15.35
	Town < 100,000 residents	181	16.44
	City < 250,000 residents	113	10.26
	City > 250,000 residents	372	33.79
Employment status	Pupil/student	418	37.97
	Professionally active	553	50.23
	Unemployed	72	6.53
	Pensioner	58	5.27
Self-reported health status	Excellent	87	7.90
	Very good	457	41.51
	Good	475	43.14
	Not too good	70	6.36
	Poor	12	1.09
Type of professional work	Blue-collar	160	14.53
	On benefits	11	0.99
	White-collar	393	35.69
Self-estimated risk of COVID-19 infection at the workplace	No risk	130	11.80
	Small risk	150	13.62
	Moderate risk	540	49.04
	High risk	281	25.54
Place of work during the pandemic	On-site work	230	20.89
	Remote work	202	18.35
	Hybrid work	121	10.99
	Unemployed	61	5.54

Abbreviations: y, years.

**Table 2:** Attitudes of Poles to restrictions during the COVID-19 pandemic.

Type of Restrictions		Number of Respondents, n	Percentage of Respondents, %
Covering the mouth and nose with a mask and/or a face shield in public places, including:	Any	1071	97.28
	Surgical masks	422	38.33
	Cotton masks	508	46.14
	Medical filtering half-masks FFP1, FFP2, FFP3	85	7.72
Covering the mouth when sneezing or coughing		985	97.55
Self-reported frequency of hand washing with soap and water	Very often	553	50.23
	Often	491	44.60
	Sometimes	51	4.63
	Rarely	5	0.45
	Never	1	0.09
Self-reported frequency of using hand disinfectant liquids with high ethanol content in public places	“I usually use them” (very often, often)	650	59.04
	“I usually do not use them” (sometimes, rarely, never)	450	40.96
Social distancing (minimum 1.5 m distance) in public places	“I usually maintain social distance” (very often, often)	923	83.83
	“I usually do not maintain social distance” (sometimes, rarely, never)	178	16.17
Self-reported use of disposable gloves in public places	Very often	48	4.36
	Often	108	9.81
	Sometimes	191	17.35
	Rarely	384	34.88
	Never	370	33.61

Abbreviations: FFP, filtering facepiece.

**Table 3:** Level of compliance with restrictions during the COVID-19 pandemic.

Level of Compliance,		Number (%) of Respondents
points (Me, Q1–Q3)	19 (17–20)	
Good	19–24	428 (38.87)
Average	15–18	528 (47.96)
Low	0–14	145 (13.17)

Abbreviations: Me, Median; Q1–Q3, upper and lower quartile.

**Table 4:** Univariate and multiple regression analysis of selected factors related to compliance with pandemic restrictions among Poles.

Variable		Level of Compliance with Restrictions					
		Univariate Model			Multiple Model		
		Coeff.	SE	*p* Value	Coeff.	SE	*p* Value
Sex	Female	(ref)					
	Male	−0.67	−1.85	0.246	−0.42	0.17	0.019
Age, y	18–24	(ref)					
	25–40	1.11	0.24	0.012			
	41–60	3.48	2.43	<0.0001	2.97	2.07	<0.0001
	>60	3.85	2.09	<0.0001	3.11	1.84	<0.0001
Education	Primary	(ref)					
	Basic vocational	2.98	0.11	0.062			
	High school	0.15	−0.79	0.754			
Place of work during the pandemic	Unemployed	(ref)					
	On-site	0.79	−2.77	0.660			
	Hybrid work	0.51	−3.30	0.792			
	Remote work	0.67	−3.39	0.743			
Risk of COVID-19 infection at the workplace	No risk	(ref)					
	Small risk	0.11	−0.88	0.816			
	Moderate risk	0.18	−1.35	0.615			
	High risk	0.27	−0.89	0.770			
Self-reported health status	Excellent	(ref)					
	Very good	1.34	0.26	0.015	1.39	0.19	0.044
	Good	1.20	0.16	0.023	1.26	0.18	0.040
	Not so good	0.56	−1.84	0.647			
	Poor	−0.17	−3.06	0.908			
SARS-CoV-2 test result	Negative	(ref)					
	Positive	0.11	−0.88	0.816			
Direct contact with a person diagnosed with COVID-19	No	(ref)					
	Yes	−0.21	−2.41	0.853			
Contact with a person suspected of SARS-CoV-2 infection or with infectious materials	No	(ref)					
	Yes	0.15	−0.89	0.770			

Abbreviations: ref, reference category; coeff, coefficient; SE, standard error.

**Table 5:** Univariate and multiple logistic regression model of the association between compliance with sanitary measures and the risk of COVID-19 among Poles.

Variable		Risk of COVID-19					
		Univariate Regression Model			Multiple Regression Model		
		OR	95% CI	*p* Value	OR	95% CI	*p* Value
Sex	Female	(ref)					
	Male	1.29	0.70–3.14	0.406			
Age, y	18–24	(ref)			(ref)		
	25–40	4.56	2.56–8.07	<0.001	4.52	2.53–8.06	<0.001
	41–60	7.84	3.95–15.58	<0.001	7.89	3.97–15.70	<0.001
	>60	3.09	0.92–10.36	0.067	3.13	0.94–10.52	0.064
Covering the mouth and nose with a mask and/or a face shield in public places	No	(ref)			(ref)		
	Yes	0.24	0.06–0.89	0.031	0.26	0.09–0.95	0.042
Self-reported frequency of hand washing with soap and water	Rarely/never	(ref)					
	Very often/often	1.22	0.11–13.50	0.876			
Self-reported frequency of using sanitizers with high ethanol content in public places	Rarely/never	(ref)			(ref)		
	Very often/often	0.45	0.30–0.68	<0.001	0.47	0.34–0.74	<0.001
Maintaining social distance (minimum 1.5-m distance) in public space	No	(ref)			(ref)		
	Yes	0.593	0.34–1.05	0.052	0.53	0.24–0.97	0.043
Self-reported use of disposable gloves in public places	Rarely/never	(ref)			(ref)		
	Very often/often	0.44	0.25–0.72	<0.001	0.49	0.36–0.83	<0.001

Abbreviations: ref, reference category; OR, odds ratio; CI, confidence interval.

## Data Availability

The datasets used and analyzed during the presented study are available from the corresponding author on reasonable request.

## Supplementary Materials

The following supporting information can be downloaded at: https://www.mdpi.com/article/10.3390/healthcare11060914/s1, File S1: The study questionnaire.
