# Supplementary material for: Association between Compliance with COVID-19 Restrictions and the Risk of SARS-CoV-2 Infection in Poland
Source: Healthcare (Basel). 2023 Mar 22;11(6):914. doi: 10.3390/healthcare11060914 (PMC10048166; doi:10.3390/healthcare11060914)
Supplement: Supplementary file 1 [file healthcare-11-00914-s001.zip › healthcare-2245910-supplementary.pdf]

File S1 The study questionnaire

"Association between compliance with COVID-19 restrictions and the risk of SARS-CoV-2 infection in Poland"

We encourage everyone over the age of 18 to complete the following questionnaire: "Association between compliance with COVID-19 restrictions and the risk of SARS-CoV-2 infection in Poland"

Dear Sir or Madam,

The Department of Internal Diseases, Metabolic Disorders and Arterial Hypertension as well as the Department of Pharmacoeconomics and Social Pharmacy of the Poznan University of Medical Sciences are conducting a survey of the public opinion regarding the association between compliance with COVID-19 restrictions and the risk of SARS-CoV-2 infection in Poland.

The survey aims to collect information necessary for a scientific analysis and to prepare a strategy for dealing with health-threatening situations.

The survey is safe, free of charge, and anonymous. Personal data of respondents shall not be recorded. Data that could be used to identify the survey participants in the future shall not be used.

Upon agreeing to participate in the survey, you will be asked to respond to questions in an online survey, which will be used to collect the required demographic information, assess a history of COVID-19 contact, COVID-19 knowledge, and preventative measures as well as the availability of health-related information with regard to COVID-19. We will also assess the impact of the pandemic on your health-related behaviors.

Every respondent may approach the authors to ask for information regarding the survey questions and to obtain a report once the survey has been completed.

Consent to take part in the survey

I have read and understood the information pertaining to the survey. By responding to this questionnaire, I voluntarily consent to participate in the survey and I am aware that I can withdraw my consent to participate in the subsequent parts of the survey at any time without providing a reason.

Pursuant to the Polish law (Personal Data Protection Act of 10 May 2018), this survey shall be anonymous.

1. **Sex: ***
- o Female
- o Male

2. **Age: ***
- o 18-24
- o 25-40
- o 41-60
- o more than 60.

3. **Education: ***
- o Primary
- o Basic vocational
- o High school

4. **Your place of residence: ***
- o Village
- o Town, up to 50 thousand residents
- o Town, up to 100 thousand residents
- o City, up to 250 thousand residents
- o City, more than 250 thousand residents.

5. **What is your professional activity status? ***
- o Pupil/student
- o Professionally active
- o Unemployed
- o Pensioner

6. **If you are professionally active, what type of work do you do? ***
- o Blue-collar
- o White-collar

7. **If you are professionally active, where do you work during the pandemic? ***
- o At a workplace
- o I work from home
- o Currently I am not working as my workplace has closed down / I have closed my business
- o Currently I am not working as my workplace has closed down / I have closed my business, and I am afraid of losing my job
- o I have just lost my job / closed my business because of the market situation caused by the pandemic.

8. **If you are professionally active, is there an increased risk of COVID-19 infection risk at the workplace? ***
- o Yes, the risk is very high
- o Yes, the risk is high
- o Yes, the risk is moderate
- o Yes, the risk is low
- o There is no such risk

9. How would you describe your current state of health? *

- o Excellent
- o Very good
- o Good
- o Not so good
- o Poor

10. Which chronic illness have you been diagnosed with (you may tick more than one option) *

  - o Obesity
  - o Coronary disease
  - o Arterial hypertension (high blood pressure)
  - o Prior heart attack
  - o Prior stroke
  - o Diabetes
  - o Chronic obstructive pulmonary disease (COPD)
  - o Asthma
  - o Airborne or food allergy
  - o Osteoarthritis
  - o Spondyloarthritis
  - o Rheumatoid arthritis
  - o Cancer
  - o Depression
  - o Other diseases (please specify):
  - o I have not been diagnosed with any diseases

11. Current body weight (kg):

…………………………………………………………………

12. Height (cm): *

………………………………………………………………….

27. Do you wear a protective mask in public areas? Please select one answer that best describes your behavior. *

- o no
- o no, because I wear a face shield
- o yes, I wear a disposable surgical mask
- o yes, I wear a medical mask (FFP1, FFP2, FFP3)
- o yes, I wear a cotton mask

28. When in public areas, do you observe the recommended social distance (a minimum distance of 1.5 meters)? *

- o Yes
- o No

Please answer the following questions based on the scale described below:

very often – it affects me very much or most of the time;
often – it affects me to a large extent or often;
sometimes – it affects me to some extent or occasionally;
rarely – it affects me to a small extent or rarely;
never – it does not concern me at all.

29. Do you cover your mouth when coughing or sneezing? *

- Very often
- Often
- Sometimes
- Rarely
- Never

30. How often do you wash your hands using soap and water? *

- Very often
- Often
- Sometimes
- Rarely
- Never

31. How often do you disinfect your hands (using a sanitizer with high ethanol content) ?*

- Very often
- Often
- Sometimes
- Rarely
- Never

32. When shopping, how often do you use disposable gloves? *

- Very often
- Often
- Sometimes
- Rarely
- Never

33. Did you experience any of the following symptoms during the COVID-19 pandemic? (You may tick more than one option). *

- Persistent fever (above 38ºC for at least 1 day)
- Shivers
- Headache
- Muscle pain
- Cough
- Difficulties breathing
- Dizziness
- Runny nose
- Sore throat
- Sudden loss of smell or taste

- I did not have any of the above symptoms

34. Were you in direct contact with a person infected with SARS-CoV-2? *
- Yes
- No

35. Were you in contact with a person with a suspected SARS-CoV-2 infection or with pathogens? *
- Yes
- No

36. Were you on compulsory quarantine? *
- No
- Yes, once
- Yes, twice
- Yes, more than twice

37. Did you undergo a COVID-19 test? *
- Yes
- No

38. What was your COVID-19 test result? *
- Positive
- Negative

39. Were you hospitalized at any time during the pandemic because of COVID-19? *
- Yes
- No

40. If you were hospitalized due to COVID-19, was it necessary apply mechanical ventilation (ventilator)? *
- Yes
- No

41. Did you suffer from any complications due to COVID-19? *
- Yes
- No
